# Supplementary material for: Impact of Alternatively Polyadenylated Isoforms of ETHYLENE RESPONSE FACTOR4 with Activator and Repressor Function on Senescence in Arabidopsis thaliana L
Source: Genes (Basel). 2019 Jan 28;10(2):91. doi: 10.3390/genes10020091 (PMC6409740; doi:10.3390/genes10020091)
Supplement: Supplementary file 1 [file genes-10-00091-s001.pdf]

Supplemental Figures

Figure S1

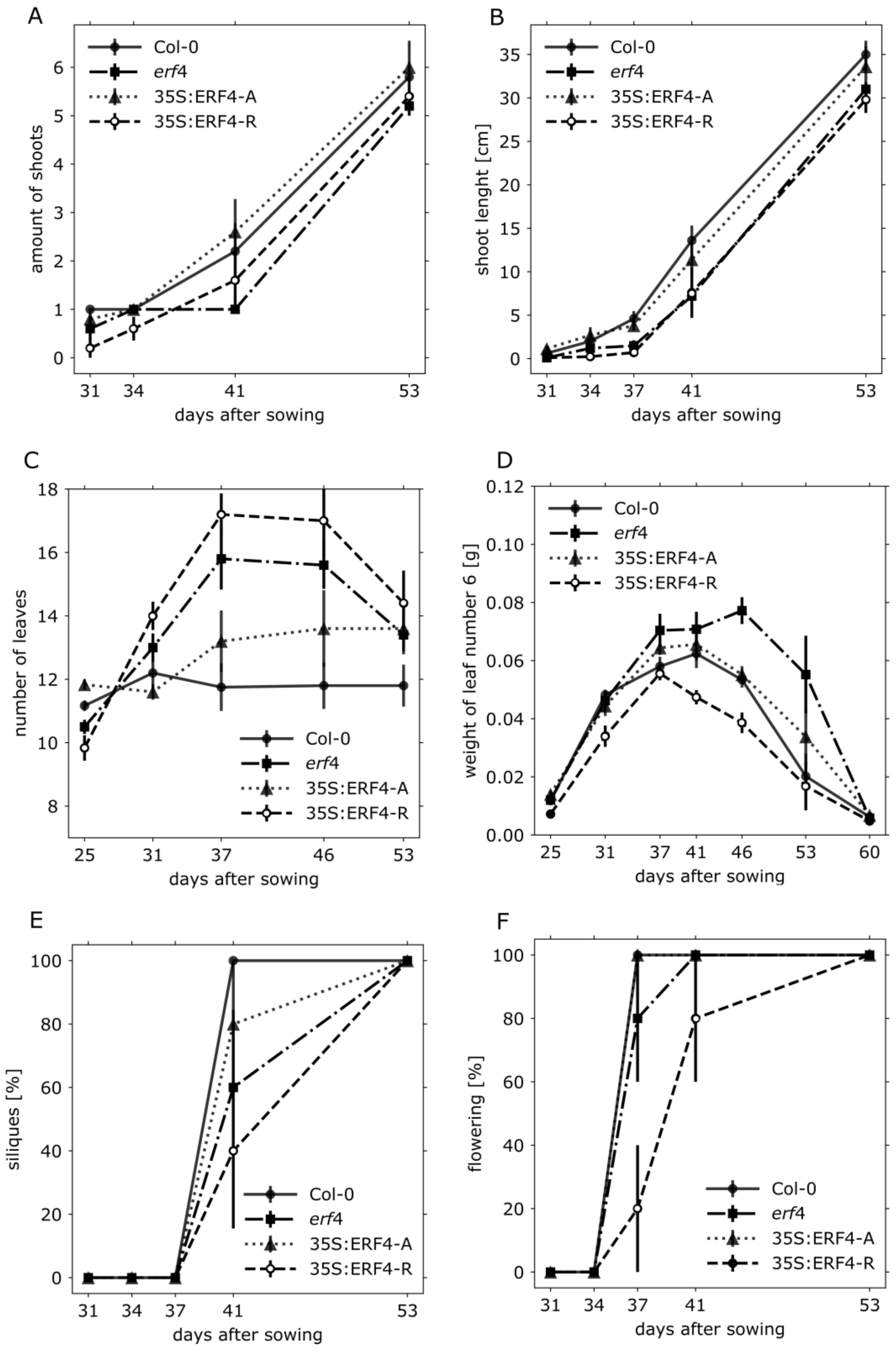

Continuation on next page

Continuation of Figure S1

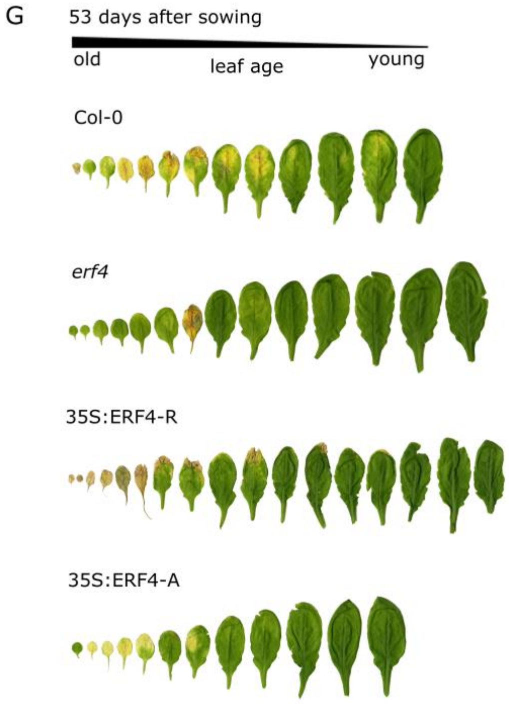

Measurement of different parameters over the development of Col-0, 35S:ERF4-R, 35S:ERF4-A and erf4 mutant plant lines. (A) Shoot number and (B) shoot lengths (C) rosette leaf number and (D) fresh weight of leaf No. 6 were analyzed and percentage of plants with (E) siliques and (F) with flowers was determined at different time points. Bolting occurred between 28 and 31 DAS. Leaf number in (C) decreases at 53 days after sowing, because some leaves were already totally decayed and not counted any more. Data are means (±SE) of 5 biological replicates. Phenotyping experiments were performed in two different plant series with similar results. (G) Representative pictures of all rosette leaves, which were sorted according to their age, of different plant lines at 53 DAS.

Figure S2

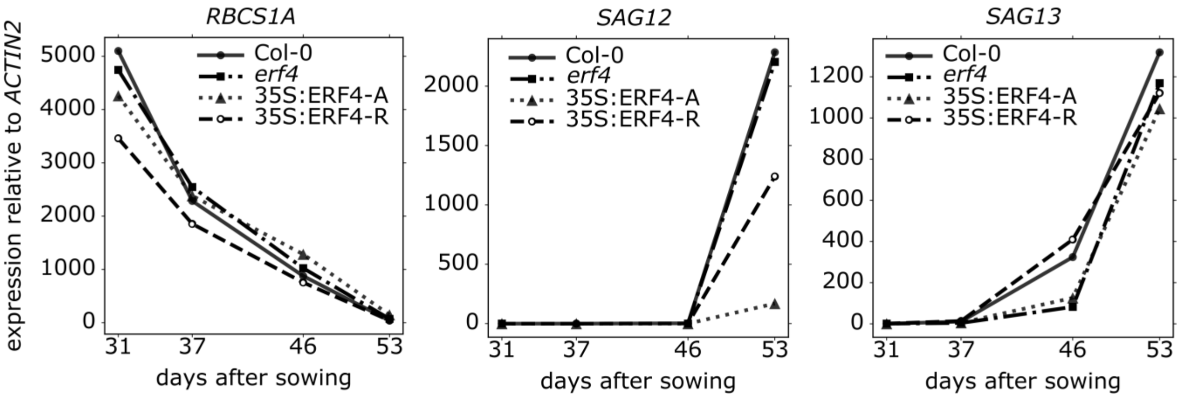

Relative expression level of senescence-related genes. qRT-PCR of senescence marker genes was performed in Col-0, *erf4*, 35S:*ERF4-A* and 35S*:ERF4-R* plants from 31-53 days after sowing for *RBCS1A*, *SAG13*, *SAG12*. Five leaves No. 7 were pooled for RNA isolation. Data are means of 2 technical replicates. Relative expression level was calculated and normalized to *ACTIN*2 based on the method by Pfaffl *[40].

*[40] Pfaffl, M.W. A new mathematical model for relative quantification in real-time RT-PCR. *Nucleic Acids Res.* **2001**, *29*, 45e.

Figure S3

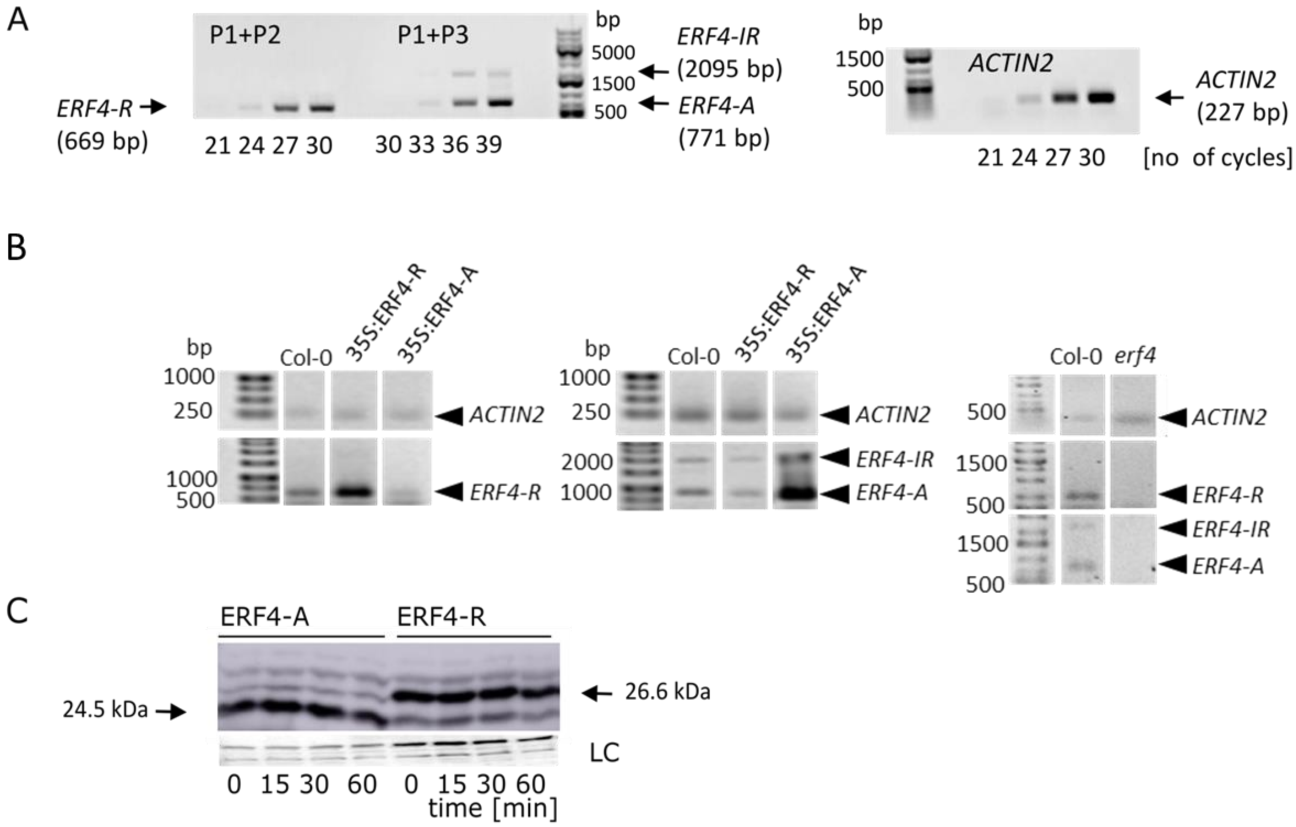

*Isoform specific semi-quantitative RT-PCR***.** (A) Determination of the exponential range of amplification for *ERF4-A*, *ERF4-R* and *ACTIN2*. The genes were amplified using a pool of cDNAs, which originated from plants of different age. The PCR reaction is visualized on a 1% agarose gel. For optimal conditions for quantification, different amounts of cDNA were used for each isoform (*ERF4-A* 3 µl, *ERF4-R* 2 µl, *ACT2* 1µl). (B) Expression of the different *ERF4* isoforms in the 35S:*ERF4-R*, the 35S:*ERF4-A*, and the *erf4* mutant line compared to Col-0; one representative example of the semi-quantitative RT-PCR is shown, expression analyses were repeated at least three times with similar results. (C) In vitro plant protein stability in bacterial crude extracts was tested by using 25 µg of bacterial crude protein extracts of *E. coli* BL21 Rosetta expressing recombinant HIS-tagged ERF4-A (24.5 kDa) and ERF4-R (26.6 kDa) proteins, respectively. Proteins were incubated for 0-60 min. In contrast to incubation with crude plant protein extracts (Figure 2D), no degradation was observed. Amido black staining of the upper region of the PVDF membranes is presented as loading control (LC).

Figure S4

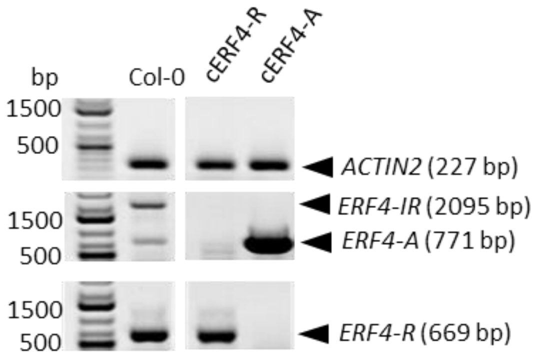

*Isoform specific semi-quantitative RT-PCR of the complementation lines***.** Representative agarose gel images of the semi-quantitative RT-PCR using RNA isolated from plants of different genotypes: Col-0, cERF4-A, cERF4-R. 36 cycles and 3 µl cDNA were used for *ERF4-A*/*ERF4-IR* and 27 cycles and 2 µl cDNA for *ERF4-R*. Experiments were repeated at least 3 times with similar results.

Figure S5

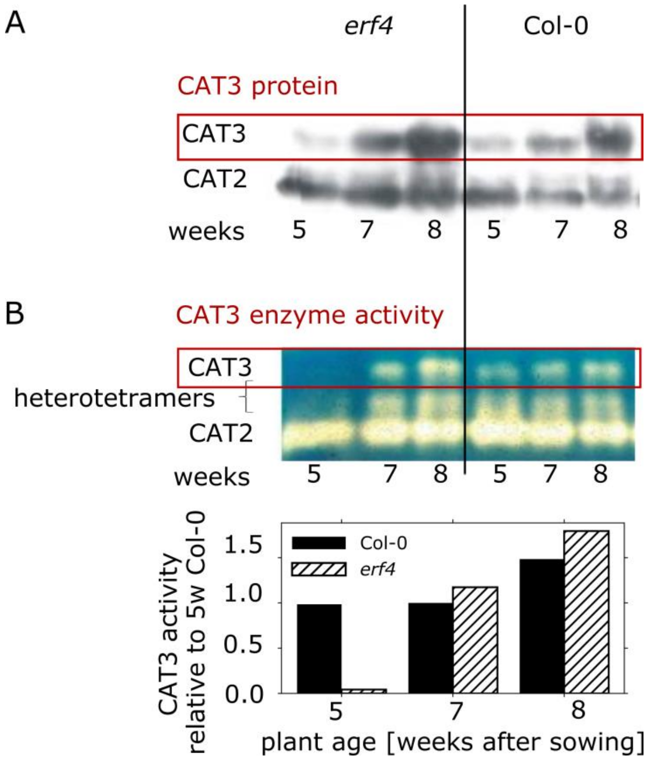

CATALASE protein amounts and enzyme activity in 5- to 8- week-old Col-0 and erf4 mutant plants. (**A**) Western Blot of 30 µg of crude protein extracts separated on a 7.5% gel with subsequent immunodetection using polyclonal anti-rye-CAT antibodies, which were kindly provided by J. Feierabend*[58]. (**B**) Enzyme activity of catalase isoforms visualized in a native zymogram using 5 µg of crude protein separated on a 7.5% native gel, Western Blotting and subsequent staining for catalase activity. Intensities of the CAT3 bands were quantified using *ImageJ*.

*[58] Hertwig, B.; Streb, P.; Feierabend, J. Light dependence of catalase synthesis and degradation in leaves and the influence of interfering stress conditions. *Plant Physiol.* **1992,** *100*, 1547-1553.

Figure S6

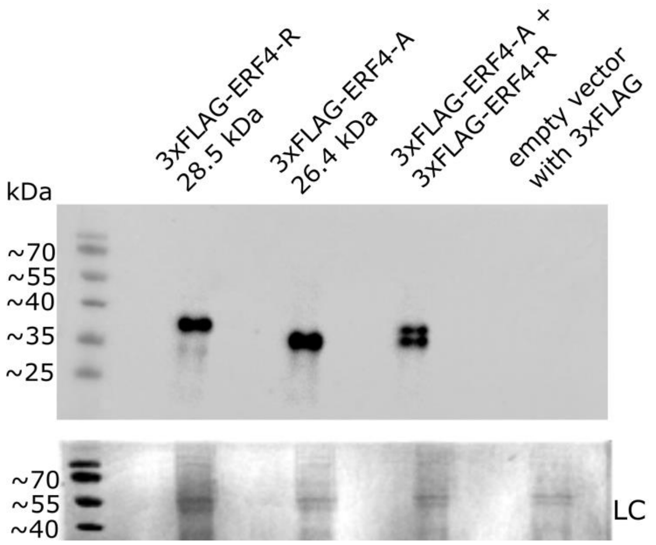

ERF4-A and ERF4-R protein expression in Arabidopsis protoplasts. Crude extracts of Arabidopsis protoplasts expressing 3xFLAG-tagged ERF4 isoforms and the empty vector with 3xFLAG were separated on a SDS gel. After Western blotting, proteins were immunodetected with monoclonal anti-FLAG primary antibodies (Sigma-Aldrich) and anti-mouse secondary antibodies (Sigma-Aldrich). As a loading control (LC), the proteins on the PVDF membrane were stained with amido black after immunodetection.

Figure S7

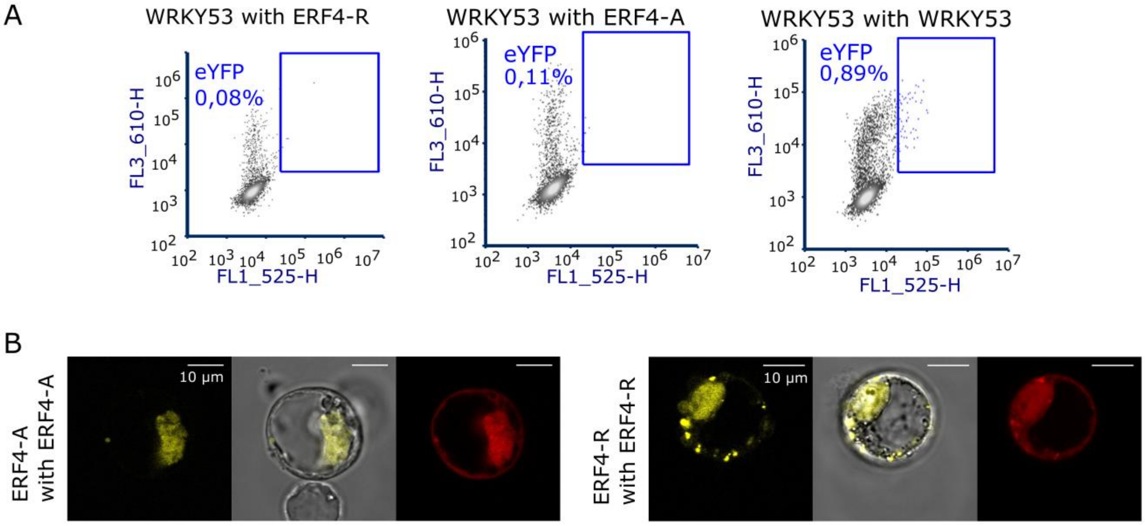

Protein-Protein interactions in transiently transformed protoplasts. (A) BiFC flow cytometry experiments were performed in Arabidopsis protoplasts co-expressing ERF4-R, ERF4-A and WRKY53 fused with YFP-N and YFP-C, respectively. Representative graphs of the flow cytometry results are shown. Blue dots represent eYFP signals of interaction. Blue squares mark the cells showing eYFP signal. (B) Confocal microscopy pictures of Arabidopsis protoplasts, transfected with the same BiFC constructs. eYFP indicates interaction; mRFP is a transfection control.

Figure S8

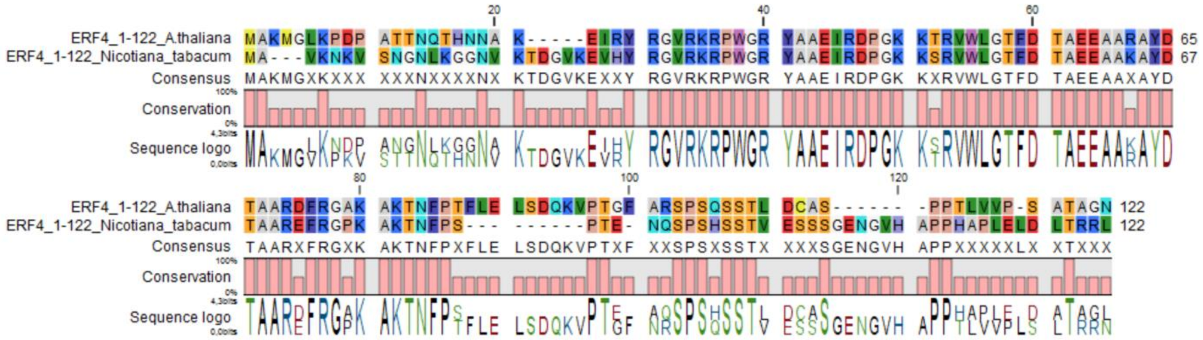

Protein alignment of the first 122 amino acids (AA) of *A. thaliana* and *N. tabacum* ERF4, 70 AA of the 122 AA (57.4%) are identical, AA at position No. 44-75 are highly conserved with 7 sour AA (indicated in red).

Table S1: *Primer sequences*

primer name	sequence	method	reference
ERF4R-qF	TTGCCTCCTCCATCGGAACAGG	qRT-PCR	Lyons et al., 2017
ERF4R-qR	CAAAAAGAAGAAGAAACGCATGCGC	qRT-PCR	Lyons et al., 2017
ERF4IR-qF	TTCCAGCAGACACGCAGCCG	qRT-PCR	Lyons et al., 2017
ERF4IR-qF	TGTCCGTACTCTGTGAGTGGACCC	qRT-PCR	Lyons et al., 2017
ERF4A-qF	GGCTTGTGGTGCCCAAAGCG	qRT-PCR	Lyons et al., 2017
ERF4A-qR	TCACACCCTCTTATACGTCGTCGT	qRT-PCR	Lyons et al., 2017
CAT3-qF	AGGTACAGATCATGGGCACCAG	qRT-PCR	
CAT3-qR	AAGGATCGATCAGCCTGAGACC	qRT-PCR	
ACTIN2-f	ACCCGATGGGCAAGTCATCACG	qRT-PCR	
ACTIN2-r	TCCCACAAACGAGGGCTGGA	qRT-PCR	
SAG12-f	TCCTTACAAAGGCGAAGACGCTAC	qRT-PCR	
SAG12-r	ACCGGGACATCCTCATAACCTG	qRT-PCR	
SAG13-f	AGGGAGCATCGTGCTCATATCC	qRT-PCR	
SAG13-r	CCAGCTGATTCATGGCTCCTTTG	qRT-PCR	

WRKY53-f	ATCCCGGCAGTGTTCCAGAATC	qRT-PCR	
WRKY53-r	AGAACCTCCTCCATCGGCAAAC	qRT-PCR	
RBCS1A-f	ACCTTCCTGACCTTACCGATTCCG	qRT-PCR	
RBCS1A-r	GGTACACAAATCCGTGCTCCAAC	qRT-PCR	
CAB1-f	TGCACTACTCAACCTCAATGGC	qRT-PCR	
CAB1-r	AAAGCTTGACGGCCTTACCG	qRT-PCR	
FPA-f	CAACCACCAGCAGATAAGGC	qRT-PCR	
FPA-r	TGTTGTACCCTGACCATCCC	qRT-PCR	
ESP/ESR-f	GTGTGGGAAAAGTTGGGAGA	qRT-PCR	
ESP/ESR-r	CATGAGGAGGCCATTCTTTC	qRT-PCR	
ERF4-Start-f	ATGGCCAAGATGGGCTTGAAACCCGA	sqRT-PCR	Lyons et al., 2017
ERFA-STOP-r	CTACACGAGAATCACGAAAGGATAGTTATTGACT	sqRT-PCR	Lyons et al., 2017
ERF4R-STOP-r	TCAGGCCTGTTCCGATGGAGGAGG	sqRT-PCR	Lyons et al., 2017
Promoter-CAT3-f	AATGCTGACTTGTCGGGGTCAGCGATTATTATTAGTCACCGAACGAATTTTTCTT	EMSA, ELISA	
Promoter-CAT3-r	AAGAAAAATTCGTTCGGTGACTAATAATAATCGCTGACCCCGACAAGTCAGCATT	EMSA, ELISA	

PW53WBox1-f (mutated)	ATGGTTTGAAAATTTAAAAAAATTTTCA	EMSA	
PW53WBox1-r (mutated)	TGAAAATTTTTTTAAATTTTCAAACCAT	EMSA	
ERF4-attB1-f	GGGGACAAGTTTGTACAAAAAAGCAGGCTTCATGGCCAAGATGGGCTTGAA	cloning	
ERF4A-attB2-r	GGGGACCACTTTGTACAAGAAAGCTGGGTTCATTGTTTTGTACCTTCGA	cloning	
ERF4R-attB2-r	GGGGACCACTTTGTACAAGAAAGCTGGGTTCAGGCCTGTTCCGATGGAG	cloning	
Promoter-CAT3-attB1-f	GGGGACAAGTTTGTACAAAAAAGCAGGCTGGGGTGAATCTAGATATCAG	cloning	
Promoter-CAT3-attB2-r	GGGGACCACTTTGTACAAGAAAGCTGGGTTGGTGATGATAGAAGGTTGA	cloning	
pERF4-3kb-f	GGGGACAAGTTTGTACAAAAAAGCAGGCTTATCGCAACCAAACTCTCTT	cloning	
pERF4-3kb-r	GGGGACCACTTTGTACAAGAAAGCTGGGTTCTCGGATAGATAGATTAGA	cloning	
attR3-ERF4-f	GGGGACAACTTTGTATAATAAAGTT GGAATGGCCAAGATGGGCTTGA	2in1 BiFC cloning	
attR1-ERF4-f	GGGGACAAGTTTGTACAAAAAAGCAGGCTTAATGGCCAAGATGGGCTTGA	2in1 BiFC cloning	
ERF4A-Stop-attR2	GGGGACCACTTTGTACAAGAAAGCTGGGTTCATTGTTTTGTACCTTCGAA	2in1 BiFC cloning	
ERF4R-Stop-attR2	GGGGACCACTTTGTACAAGAAAGCTGGGTTCAGGCCTGTTCCGAT	2in1 BiFC cloning	
ERF4R-Stop-attR4	GGGGACAACTTTGTATAGAAAAGTTGGGTTCAGGCCTGTTCCGAT	2in1 BiFC cloning	
ERF4A-Stop-attR4	GGGGACAACTTTGTATAGAAAAGTTGGGTTCATTGTTTTGTACCTTCGAA	2in1 BiFC cloning	
attR3-WRKY53	GGGGACAACTTTGTATAATAAAGTTGGAATGATGGAAGGAAGAGATATGTTA	2in1 BiFC	

	AGTT	cloning	
attR1-WRKY53	GGGGACAAGTTTGTACAAAAAAGCAGGCTTAATGATGGAAGGAAGAGATATGTTAAGTT	2in1 BiFC cloning	
WRKY53-attR2	GGGGACCACTTTGTACAAGAAAGCTGGGTTTAATAATAAATCGACTCGTGTAAAA	2in1 BiFC cloning	
WRKY53-attR4	GGGGACAACTTTGTATAGAAAAGTTGGGTTTAATAATAAATCGACTCGTGTAAAA	2in1 BiFC cloning	
Cat3-8 eco	ggaattccGAGCAATGCTGACTTGTCG	Cloning for Y1H	
		Cloning for Y1H	
Cat3-19-xba	ctctagagACATGTTCGATCTTATCGCA		
